# A Review of Glymphatics and the Impact of Osteopathic Manipulative Treatment in Alzheimer's Disease, Concussions, and Beyond

**DOI:** 10.7759/cureus.23620

**Published:** 2022-03-29

**Authors:** Maxwell A Marino, Sarah Petrova, Raed Sweiss, Jason Duong, Dan E Miulli

**Affiliations:** 1 Neurosurgery, Riverside University Health System Medical Center, Riverside, USA; 2 Neurosurgery, California University of Science and Medicine, Colton, USA; 3 Neurosurgery, Riverside University Health System Medical Center, Moreno Valley, USA; 4 Neurological Surgery, Arrowhead Regional Medical Center, Colton, USA; 5 Neurosurgery, Arrowhead Regional Medical Center, Colton, USA

**Keywords:** mechanical lymphatic drainage, cerebrospinal fluid biomarkers, intracranial pressure, cellular aging, alzheimer’s dementia, chronic traumatic encephalopathy, brain concussion, space travel, glymphatics, osteopathic manipulative treatment (omt)

## Abstract

Glymph is a fluid that circulates in the brain interstitium and, under pathological conditions, unusually accumulates and enhances the buildup of other noxious molecules. The study of this process of circulation, accumulation, and clearance is called glymphatics. We review the physiology of glymphatics and then dive into recent innovative research surrounding this neurological field of study and how it has applied to mainstream pathological processes, including Alzheimer's disease and spectrums of traumatic brain injury that range from a concussion to chronic traumatic encephalopathy (CTE). Furthermore, we explore the implications of glymphatics and a new and developing frontier of healthcare in space travel; with the advent of a Space Force and the introduction of space travel to consumer markets, this is an exciting time to develop novel techniques in enhancing its safety and optimizing human physiology for best outcomes. Therefore, we also propose that osteopathic manipulative treatment (OMT) plays an intuitive role in the treatment of abnormal glymphatics, as adjunctive therapy in Alzheimer's and CTE, and as a future staple before, during, and after space travel for the benefit of both enhancing healthcare in chronic conditions and advancing the capabilities of the human race in its shining new endeavor.

## Introduction and background

This review focuses on the connection between concussions, Alzheimer’s disease (AD), and brain edema on glymphatic neurophysiology. Osteopathic principles have been applied to these disease processes to better understand and treat symptoms. Understanding glymphatic anatomy and physiology may explain some of the underlying pathophysiology of these clinical conditions. We also review the current evidence that can lead to enhancement of the glymphatic functioning in brain edema to restore and improve human health. This review may provide context for the practitioner to utilize with patients when educating them about neurophysiology, glymphatic disease processes, and treatment plans to help improve outcomes.

## Review

Concussions

Concussions are extremely common in the field of sports. A concussion is defined as a mild traumatic brain injury (TBI) that can result in symptoms such as loss of consciousness, amnesia, and even as minor as confusion. Its annual incidence is estimated to be as high as 0.6% of the US population [1]. Loss of consciousness only occurs in approximately 10% of concussions. Failure to identify that concussions may occur in the absence of loss of consciousness may yield to underreporting [2]. Furthermore, concussions lead to both acute and chronic cognitive and behavioral changes. These changes are brought about by changes at the cellular level. Each successive concussion has an additive effect, causing even more damage.

Understanding the connection between the neuropathology and neurophysiology of concussions as well as between the structure and function can help mitigate the suboptimal outcome. In concussions, percussive shock waves transmit forces through brain tissues of different densities. The kinetic energy from this injury will be absorbed in the CSF, gray matter, and white matter. The mechanical energy not absorbed by the CSF is transmitted throughout the brain tissue, disrupts the cytoskeletal architecture, impairs organelle structure and function, and ultimately affects the biochemical function of the brain. This cascade interferes with the function of neurotransmitters [3].

The early release of neurotransmitters, including glutamate, can cause excitatory neuronal toxicity, leading to the accumulation of additional metabolites and byproducts. This leads to neurologic symptoms including amnesia or confusion if the deleterious metabolites are not broken down and cleared through the brain’s normal glymphatic functions. This neurotransmitter release and cell injury lead to dysfunction of electrochemical signal transduction, which has been demonstrated on quantitative EEG. These changes disrupt the blood-brain barrier (BBB), leading to excess fluids in the brain interstitium. TBI further impairs signal transduction, as damaged oligodendrocytes that support the brain neurons can no longer myelinate damaged axons. The rotational force of the concussion shears the white matter tracts, resulting in diffuse axonal injury and the formation of axonal retraction balls visible within deep subcortical structures on fluid-attenuated inversion recovery (FLAIR) MRI imaging. This cytological injury, when severe enough, can result in cytotoxic and vasogenic edema which can be present in patients after recovery from their concussion and severe TBI [4]. Grossly visible clinical changes may not be seen acutely in the patient with mild TBI. However, the pathologic changes and damage to the white matter tracts can be seen on magnetic resonance angiography (MRA) and diffusion tensor imaging (DTI), even if symptoms no longer persist [5].

Even in mild TBI, if the concentration of abnormal cytotoxic byproducts is too high, the parenchyma may not appropriately recover to the patient’s functional or structural baseline. The release of nicotinamide adenine dinucleotide phosphate (NADPH) oxidases contributes to the damage to the BBB at nearby sites. A recent study identified a novel blood biomarker called P13BP, a selective substrate of the neurodegenerative protein Calpain-2, that is directly related to the severity of TBI. P13BP levels were slightly elevated six hours after TBI, peaked at 24 hours, and were still significantly elevated seven days after TBI [6]. They returned to baseline levels by 10 days after TBI, as the Glasgow coma scale (GCS) returned to baseline. The normal functions of the CNS are disrupted, furthering the cascading anatomical and physiological effects of mild TBI. If the natural compensatory responses are left unchecked, or if the structure is not restored, then secondary brain damage may occur due to detrimental effects of the inflammatory cascade and failure to clear the metabolites and byproducts from the brain interstitium after a concussion.

Symptoms after a concussion can last up to four weeks or longer, and the deleterious effects of a concussion can also lead to post-concussion syndrome (PCS). The symptoms of PCS may persist for a great length of time, leading to long-term disability. However, PCS may be underdiagnosed if the head trauma is minor and is overlooked by the patient, family, or healthcare provider. The diagnosis of PCS is further challenged by its vague or “normal” everyday symptoms, including confounding life factors. While PCS can begin shortly after injury, it is usually delayed and can persist for many months. Further confirming the diagnosis and effective testing for PCS is limited [3].

Testing for TBI is being investigated to assist in diagnosis and to better understand the disease processes. One such test is the quantitative electroencephalogram (qEEG), which can be used during both awake and sleep states. Patients with concussions demonstrate less awake alpha wave activity and more awake theta and delta wave activity, and, while asleep, a longer rapid eye movement (REM) stage. Changes in qEEG can be detected in mild TBI both immediately in acute injury and up to one year after injury. The degree of changes on qEEG correlates with the intensity of active symptoms. As a result, concussions can change the electrical function of the brain, which is controlled by the chemical nature and structure of the brain [7].

The first serum marker approved by the FDA to test for TBI identifies the presence of ubiquitin carboxy-terminal hydrolase-L1 (UCH-L1) and glial fibrillary acid protein (GFAP) in CSF. Ubiquitin is a neuron-specific protein throughout the CNS, serving as an antioxidant. It scavenges the brain for toxins and moves the products along and out of the brain fluid highway, called the glymphatics, to decrease long-term injury. GFAP is found in CNS astrocytes and ependymal cells, and as a filament protein, it provides support and strength to cells. The delayed changes seen on DTI after mild TBI may be directly related to the loss of this GFAP protein [8,9]. In concussions and TBI, this loss of signal translates to an increased serum concentration GFAP that can be measured. As these breakdown products collect, there is a backup of toxic metabolites causing further damage to the brain.

Additional biomarkers to evaluate concussions are the calcium-binding protein S100B, and cytokine CCL11. S100B is released from the astrocytes cytoplasm and crosses the broken BBB. CCL11 is a protein inflammatory marker found to be elevated in the CSF of patients with chronic traumatic encephalopathy (CTE). Neurofilament light polypeptides, providing axonal structural support, has also been detected in the CSF of patients with TBI. As discussed previously, another such biomarker, Calpain-2, has been found to cleave PTPN13 into several breakdown products, including P13BP, leading to inflammation, activation of astrocytes and microglia, axonal degeneration, and Tau protein phosphorylation in TBI. These microscopic changes lead to a breakdown of the structure and an increased concentration of metabolites and byproducts in the brain parenchyma that may not be cleared appropriately [6]. Again, we see a possibility of a buildup of toxic metabolites due to lack of clearance due to damage to the normal pathways.

Chronic traumatic encephalopathy

Repetitive brain injury has long-term consequences that are directly linked to poor outcomes for patients. The development of CTE is one of those consequences. CTE can take several years for its progressive effects to become evident, sometimes only after cognitive and psychiatric impairments significantly interfere with a patient's life and their ability to work resulting in an unemployment rate 60% higher than the general population. Further symptoms include increased rates of depression and 3-4 times increased rates of suicide than the general population [3].

The pathological manifestations of CTE, demonstrated at autopsy, include accumulation of neurofibrillary tangles, Tau protein deposition, and a histological appearance very similar to patients with chronic AD. CTE is not the only manifestation of repeat TBI. Many patients, particularly as demonstrated in boxers, have developed a Parkinsonian-like disease attributed to blunt force trauma to the head. These individuals demonstrate higher incidences of depression, anxiety, and psychosis [3]. Similar to concussions, CTE continues to highlight this concept of a buildup of toxic metabolites in the brain.

Alzheimer’s disease

Similar to CTE, AD is affected by the failed clearance of the brain's interstitial glymphatic system. It is a neurodegenerative brain disorder and a common cause of dementia in the elderly secondary to the accumulation of tau protein deposition in the brain.

The prevalence of mild cognitive impairment in adults aged ≥65 years is approximately 20% in the USA. This risk increases with age and sex, with a predilection for men. Mild cognitive impairment is considered the onset of symptoms before dementia. No medications have been approved for the effective treatment of this mild cognitive impairment. It is also unclear which people with mild cognitive impairment patients will progress to severe dementia. The global brain health initiatives provide a list of exercises that may help in prevention and progression. However, this is not specific to AD. Although patients with mild cognitive impairment are at greater risk of developing dementia, that risk estimate varies anywhere from 5 to 20%. Current best estimates are that 10% of seniors with mild cognitive impairment will progress to AD [10]. One biomarker for AD measures amyloid-beta (Aβ) deposition. This biomarker tells us that there was a change in brain structure anatomy and physiology at the cellular level, which leads to functional brain changes at the personal, familial, and societal levels. Individuals with mild cognitive impairment that progress to dementia also demonstrate hippocampal atrophy on MRI [10].

Although the pathological progression of AD varies, the initial development of neuritic plaques and neurofibrillary tangles in the medial temporal lobe, a region that is phylogenetically older than the neocortex, is universally present. These same types of neuritic plaques and neurofibrillary tangles are also seen in CTE. While CSF clears interstitial spaces of the brain of solutes and byproducts, the patterns of CSF clearance are different. As CSF in the brain interstitial spaces flows from the neocortex along this posterior route, it leaks out of the CNS along with the dural sheathing that wraps cranial nerves and spinal nerves. A minority of the neocortex CSF arrives in the lumbar cistern at the base of the spine. However, this CSF is composed differently from other areas of CSF throughout the brain and spinal cord because of the solutes that it picks up, as it absorbs from passing different structures with different metabolic and chemical functions [11].

Most CSF that clears from the older allocortex of the medial temporal lobes and the basal forebrain of the anterior fossa drains along the entorhinal cortex, picking up apoptotic debris and insoluble metabolites. This specific CSF flows anteriorly toward the anterior frontal pole, running along a loosely packed fiber bundle called the lateral olfactory stria, which connects to the olfactory trigone on the inferior surface of the basal forebrain. This region contains cholinergic neurons that are critical for memory formation. It then proceeds anteriorly along the olfactory tract to the olfactory bulb and fibers through the cribriform plate to the upper nasal cavity. As with cranial nerves and spinal nerves, the olfactory nerve is surrounded by meshes that include conduits for brain interstitial fluid to flow outward from the CNS [12,13].

A preprint article, which is not yet peer-reviewed, investigated the cribriform plate morphology in healthy and Alzheimer’s diseased individuals [14]. Using ferrets, their olfactory drainage through the cribriform plate was sealed off. When compared to normal ferrets, these ferrets without appropriate drainage developed moderate cognitive impairment. Since apertures of the cribriform plate are conduits for CSF to flow along olfactory nerve fibers from the olfactory fossa to nasal submucosa, it was hypothesized that reductions in the number, size, location, and total cross-sectional area of these apertures may affect CSF outflow capacity of the cribriform plate. It was further hypothesized that a neurological and psychiatric disease, such as AD, is exacerbated by changes in the cribriform plate morphology that affect CSF egress. CT scans and deep learning were used in Project Cribose to evaluate the cribriform plate morphology in humans. Patients' memory was assessed and scans were compared to post-mortem samples to attempt to predict the onset of AD before cognitive decline [14].

This study highlights the relationship between the cribriform plate morphology and cognitive impairment, which is similar to the one seen in ferret models. As patients age, the amount of CSF flux through the cribriform plate decreases. Subjects with a confirmed post-mortem diagnosis of AD had the smallest CSF flux capacity. This relationship suggests that enhancing CSF outflow and clearance of metabolites and byproducts along the olfactory conduits and cribriform plate may mitigate the development of AD [14].

Space travel and brain edema

Space travel leads to changes in gray matter and CSF spaces in astronauts. A study of MRI scans of astronauts showed decreased gray matter volume but no changes in white matter. Some of these same areas of atrophy resemble the same morphology as in AD. After returning to the Earth, the reduction in gray matter volume appeared to improve as time passed. Additionally, there was a near-normal return of the CSF volume in the ventricles after returning. He also had normal lumbar puncture opening pressures after returning [15].

Changes in the brain CSF volume may be reflected through changes in the globe of the eye as studied over the last 40 years of space flight. One study investigated the changes in parameters involving the eye and intraocular measurements and postulated that compartmentalization and decreased venous compliance led to a shift in CSF dynamics, resulting in increased intracranial pressure (ICP). Although not measured in space, recent invasive measurements of ICP, through the placement of an external ventricular drain during parabolic flight, suggest that there is an inability to reduce ICP and CSF brain interstitial outflow. The lack of gravity in space alters these changes in a way that is not necessarily like Earth. On Earth, neurointensivists can affect ICP reduction using gravity by the head of the bed elevation to 30-45°. In space, however, the overall lack of gravity and lack of normal daily maneuvers that change CSF pressures may lead to low-level elevation of ICP [16]. There are no known ICP measurements of astronauts before or during space flight.

Alternatively, another observation showed that the changes in ocular dynamics may not be linked to increased ICP. An astronaut returning from spaceflight had unilateral grade 1 disc edema, but had a normal opening pressure via lumbar puncture 8 days after the mission [16]. In the eye with disc swelling, this astronaut had a unilateral loss of spontaneous venous pulsations (SVPs), which were previously visible. Twenty-one months after he returned to Earth, this was still absent, which suggests a more chronic rise in the optic nerve sheath pressure.

Again, this may highlight a dysfunction in the normal clearance pathways of metabolites in the brain. The normal pathway may need to be restored to prevent downstream effects of the buildup of these materials.

Overview of glial cells

Glial cells support the nervous system. They are smaller than neurons but vastly outnumber neurons. They do not contain axons or dendrites. They are vital pieces of growth, development, and recovery of the CNS [17]. Glial cells, including astrocytes, microglia, and oligodendrocytes (or Schwann cells), are found in both the CNS and peripheral nervous system (PNS). Astrocytes are involved in neuronal signaling. They also maintain brain homeostasis regarding ion concentration and solute transport. They also function in the formation of synapses.

Astrocytes are in both gray and white matter. As discussed previously, GFAP is the main component of astrocytic intermediate filaments. As the brain structure is disrupted, GFAP is released more during an injury and gets into the bloodstream. The astrocytes react to brain injury by increasing in number and changing their morphology and role after stroke, degenerative diseases, hepatic encephalopathy, and other injuries. Furthermore, as previously discussed, markers of astrocytic injury, GFAP, can be measured in patients with TBI. Astrocytes and other glial cells change to account for the needs at the time. Glial cells then clear the metabolites, edema, and byproducts from the brain interstitium via the glymphatics [18].

Glymphatics

Throughout the body, except the brain, the primary concept underpinning the traditional understanding of lymphatic organization is that of a parallel system of vessels to blood vessels acting as a collecting system for material expelled from the blood vessels into the body tissue interstitium. The combination of hydrostatic pressure and oncotic force drives blood components into the body tissue interstitial space by way of fenestrated capillaries. There are no fenestrated capillaries in the brain. As the pressure created by the material in the body tissue interstitial space increases, overlapping junctions between the lymphatic epithelial cells open and allow egress of body tissue fluids into lymphatic channels. This idea that fluid and other solutes can move between spaces like this includes the mechanisms of diffusion and bulk flow, each mediated by different forces [19].

In addition to the lack of separate lymph vessels, the brain capillaries are markedly different. Specifically, systemic capillaries are leaky, given their expression of aquaporin 1 channels and their fenestrated structure. In marked contrast, the capillaries of the brain do not express aquaporin 1 channels and the relatively impermeable tight gap junctions hold the endothelium together. There is, however, a perivascular environment in each system, which plays a crucial role in fluid-handling and waste removal [20].

The brain cells surrounding the blood vessels are mostly impermeable to water and greatly impermeable to almost every other substance in the blood. This is the basis of the BBB. There is, however, unrestricted transport across the BBB for small molecules and hydrophobic compounds. These substances and molecules made inside the interstitium of the brain must be cleared, along with other compounds via the CSF compartment. They are ultimately eliminated into the bloodstream through peripheral lymphatics and arachnoid granulations. The distances between areas of brain tissue and CSF compartments are too great for efficient clearance by simple diffusion. This distance limits the clearance of larger molecules [12,13].

The brain is unique in that it does not contain a conventional lymphatic system. Peripherally, the lymphatic system can assist. In the brain, however, no specialized structure facilitates lymphatic clearance [13].

The brain contains some perivascular spaces for the fluid collection called Virchow-Robin spaces. These perivascular extensions of the pia mater surround the perforating arteries and veins along the lenticulostriate arteries and medullary perforating arteries in the basal ganglia and cerebral peduncles and make a space that enlarges with age. These are not connected with the subarachnoid space, and the fluid within each space has a different chemical composition when compared to CSF. There is water influx via the aquaporin-4 (AQP-4) channel into the peri-capillary Virchow-Robin space (VRS) [21,22].

The main brain interstitial flow from the arteries is dependent upon the glial cells to move the fluid to its outflow of the veins, dura, or ventricle. This glial vascular pathway approximates the systemic lymphatic system. This glial vascular flow is part of a system that compensates for the lack of a true lymphatic system. It has a system dynamic virtually identical to systemic lymphatic circulation. However, it works very differently. The systemic circulation has fenestrated capillaries, which leak the intravascular fluid into the tissue, creating the appropriate interstitial fluid motion into the lymphatics [13].

This glial-dependent pathway is homologous to the peripheral lymphatic system. Thus, the term the glio-vascular pathway or the “glymphatic system” was coined. Glymphatics are a pathway facilitating the clearance of interstitial solutes, metabolites, edema, and byproducts from the brain into the veins, ventricles, and spaces around the dura. This pathway consists of a peri-arterial CSF influx route, a peri-venous interstitial fluid clearance efflux route, and a transparenchymal pathway via the astrocytic AQP-4 water channel. Over 40% of subarachnoid CSF enters the brain parenchyma along perivascular spaces and then follows the arterial basement membrane to reach the basal lamina. Clearance of interstitial fluid is restricted to groups of large-caliber draining veins [21,22].

Aquaporins and their role in glymphatics

Aquaporins, of which there are at least seven in the mammalian CNS, function as bidirectional water-selective channels. The astroglial AQP-4 water channel is expressed in the perivascular astrocyte foot process, leading to perivascular CSF influx and interstitial fluid clearance pathways. The bulk of the fluid flows through these pathways. About 70% of CSF influx goes through the astrocytic AQP-4 channels. There is a more nuanced arrangement of fluid flow contingent on not only expression of AQP-4, but also a differential expression of different varieties of aquaporin, state of the glial tissue, and neurohumoral upregulation and/or downregulation [21,22].

AQP-4 is primarily distributed along astrocyte foot processes at the basement membrane, peri-capillary side of the pia mater, and glial side of the ependymal cell membrane. Essentially, all AQP-4 channels in the brain are expressed on membranes inside the BBB. As such, AQP-4 channels are only involved in water movement inside the BBB [21,22].

AQP4-dependent flow occurs alongside the glymphatic system. The CSF influx along the para-arterial pathway and the paravenous route facilitates the clearance of interstitial solutes from the brain parenchyma [19].

There is an increase in AQP-4 channels to help regulate increased brain interstitial edema associated with brain tumors. The two main routes of transporting water across the BBB are: non-specific permeability across the plasma membrane and claudin-2 at the epithelial tight junction. Although the plasma membrane has a small degree of water permeability, the amount is dependent on its lipid composition. AQP-4 channels are not at the BBB, but AQP-4 is abundantly expressed inside the brain interstitium, as discussed earlier [21].

Glymphatics and aging diseases

Glymphatics change with age and disease. Failure of the clearance of amyloid β (Aβ) may be the underlying cause of deposition of plaques leading to AD. Soluble Aβ is one of the solutes cleared from the brain interstitium using both the glymphatic pathway and through the AQP-4 channel. Brain injury affects the glymphatic pathway and is characterized by changes in astrocyte characteristics. A low-intensity injury such as concussions frequently results in long-lasting changes to the glymphatic pathway. In mild TBI, this was seen for at least one month after injury, even without tissue destruction. After ischemic or TBI, AQP-4 expression is typically elevated demonstrating increased glymphatic function to clear metabolites, edema, and byproducts [21].

Since perivascular AQP-4 plays a critical role in the clearance of solutes, changes in the AQP-4 channels after a diffuse injury can lead to the progression of dementia in TBI. With age, there is mislocalization of AQP-4 channels from the perivascular astrocytic end foot to the astrocytic neuronal body, which prevents efficient water transfer. This leads to a failure of interstitial solute clearance causing the accumulation of neurotoxic metabolites, such as amyloid β, extracellular, and intracellular cytotoxic protein aggregates [10]. These are the hallmarks of AD and CTE. In this way, reactive gliosis, by disrupting interstitial waste clearance, may be a key target for therapeutic intervention to enhance the clearance of glymphatics through several pathways.

Optic nerve sheath diameter

The eye is the extension of the brain. The optic nerve is wrapped by the optic nerve sheath that contains CSF. Changes in optic nerve sheath diameter (ONSD) are related to changes in ICP due to direct communication with the CSF within the brain. Functionally, this explains why prolonged elevations lead to optic disc edema. This increase in ONSD during ICP elevation was described by Maissan et al. in an observational study of patients suffering from TBI [23]. Barring any obstruction in the normal CSF flow, the ONSD will directly reflect changes in the ICP.

In a 2019 paper in *Surgical Neurology International*, non-invasive measurement of ICP was performed using ONSD [24]. Ocular ultrasonography was able to reliably predict ICPs or intracranial pathology by ONSD. The perineural space within the sheath contains about 0.1 mL of CSF with room to expand should the ICP increase. The trabeculations are sparser in the anterior segment of the optic nerve sheath than the posterior segment. Thus, the anterior optic nerve sheath possesses the maximum capacity to distend compared to the posterior portion. Patients in the study had enlarged ONSD reflecting an increase in ICP confirmed by direct invasive monitoring. This can account for the changes seen in space travel, which can result in optic nerve sheath distension, optic edema, and fluid shifts.

Pupillometry has also been correlated with ICP and outcomes in TBI. One of the first clinical changes seen in increased ICP can be changes in pupil reactivity, due to the pupillomotor fibers’ increased sensitivity because of their location on the periphery of the oculomotor nerve. If left untreated, more permanent pupillary changes occur, which marks the progression of the severity of the TBI. The neurological pupil index (NPi), a measure of pupillary reactivity, is scaled into three tiers: normal (3.0-4.9), abnormal (<3), and non-reactive (0). In a prospective analysis, Chen et al. found that the NPi was an early predictor of ICP changes [25].

Osteopathic manipulative treatment to improve intracranial pressure

Elevated ICP is common in severe head injuries. Overwhelming evidence supports that severely brain-injured patients with an elevated ICP are directly associated with a diminished neurological outcome often resulting from decreased cerebral perfusion pressures (CPPs) [26,27]. Aggressive management of ICP and CPP using multiple modalities, including osteopathic manipulative treatment (OMT), can improve outcomes and, consequently, affect the lives of patients and their families.

In a study from *The Journal of the American Osteopathic Association (JAOA)* in 2010, OMT was demonstrated to be safe in patients with a severe head injury and increased ICP [28]. In this cohort study, OMT techniques led to a mean decrease in ICP and a concurrent increase in mean CPP values. In a follow-up study by Dreyer et al., these techniques again found favorable trends in ICP, CPP, and outcomes when using OMT [29]. Patel and Sabini demonstrated that cranial OMT was a safe and effective adjunct in the treatment of concussions [30].

As discussed before, the majority of CSF is absorbed into the venous circulation via arachnoid villi; another significant source of CSF outflow is the brain glial lymphatic system. Improved glial lymphatic outflow can be achieved by using OMT lymphatic techniques to increase CSF pulsatility through leptomeningeal sheaths, along perivascular spaces, and across the ventricular and cisternal spaces. This would improve the clearance of cerebral waste products and edema. Therefore, the increase in CSF outflow promoted by OMT would help clear waste products and reduce edema. However, care needs to be taken when performing OMT in TBI to avoid manipulation of any injured part of the head or neck. Jugular venous compression should be avoided as well, as it increases intracranial pressure.

In a review of the CV4 technique, Jakel and von Hauenschild found improvement of autonomic function as well as the reduction in pain and sleeping patterns as observed on EEG [31]. These same types of treatments can be utilized in the patient with a concussion to similarly enhance glymphatic clearance for return to play. Additional benefits may be seen when utilized to remove interstitial brain edema such as seen in extended space travel where there is an absence of normal gravity-induced hydrostatic pressure, resulting in a venous pooling in the head and neck similar to low glymphatic flow seen in the common terrestrial brain injury. Although CSF enhanced clearance has been demonstrated from the brain, these techniques may improve those conditions associated with decreased glymphatic clearance such as AD, migraines, and strokes. This was demonstrated by Iliff et al. in 2012, linking impaired glymphatics to a dysfunctional aquaporin-4 channel in those with AD [10].

Another study investigated the direct effects of OMT on ICP and ONSD. This study found significant improvements in ICP, ONSD, and NPI after OMT, which is hypothesized to be secondary to improved glymphatic functions [32]. These treatments optimized both lymphatic flow and drainage, which may decrease ICP by improving the flow of the glymphatic system.

Overall, all these studies show the safety, along with links to efficacy, of OMT in patients' lymphatic drainage. This would help remove the buildup of toxic metabolites that occurs in these disease processes. OMT is, therefore, an effective tool to help treat these patients.

## Conclusions

The brain is unique. Fluid circulating in the brain interstitium unusually accumulates there under pathological conditions. The aquaporin channels can regulate the flow inside the brain interstitium, which can rid the brain of metabolites, edema, and byproducts. However, that fluid backs up during disease, whether due to anatomical changes, downregulation of aquaporin channels, the buildup of toxic metabolites, or the inability to naturally clear the interstitial fluid. OMT can be used to promote the transit of these fluids and materials to clear this buildup of metabolites. Understanding applications of osteopathic principles to optimize glymphatics may be applied in understanding and management of neurodegenerative diseases, concussions, effects of space travel, AD, and other causes of cerebral edema.
